# Supplementary material for: Collaborative intelligence in AI: Evaluating the performance of a council of AIs on the USMLE
Source: PLOS Digit Health. 2025 Oct 9;4(10):e0000787. doi: 10.1371/journal.pdig.0000787 (PMC12510544; doi:10.1371/journal.pdig.0000787)
Supplement: S2 File — (ZIP) [file pdig.0000787.s002.zip › S2 Data Raw data files containing unprocessed question inputs and LLM outputs including transcripts of deliberations/STEP3_Question-17-1_Type-MC-NJ.docx]

**Facilitator** **[Round 1]:** A 20-year-old man comes to the office at the request of his family for an examination 1 day after a motor vehicle accident for which he was at fault. The patient was arrested for the third time after police discovered he had been driving under the influence of alcohol. He has been your patient since early adolescence, and he has a history of truancy, shoplifting, and two attempts to run away from home. He dropped out of high school in his senior year. He was fired from his most recent job because he threatened a coworker with a hammer. He has been unemployed for the past 8 months, and as a result he has many unpaid debts. He seems unconcerned about his current difficulties and has no plans for seeking employment or paying his debts. Despite these facts, you find the patient charming and interactive, and he conveys a sincere intention to change his behavior. Which of the following is the most likely diagnosis? (A) Antisocial personality disorder (B) Borderline personality disorder (C) Conduct disorder (D) Narcissistic personality disorder (E) Schizotypal personality disorder

**AI_D:** RESTATING THE QUESTION: The inquiry is centered on determining the most probable psychological diagnosis that fits the given symptoms portrayed by a 20-year-old male. He exhibits a pattern of irresponsible behavior such as driving under influence, alienating himself from societal norms, and presenting aggressive behavior. Along with this, he's currently without employment, ignoring monetary commitments and seems indifferent towards his current situation. On the other hand, he appears agreeable and interactive, expressing an intention to modify his behavior. PLANNING: The question is a multiple-choice one, with five possible conditions that may account for the patient's behavior. The disorders suggested include Antisocial personality disorder, Borderline personality disorder, conduct disorder, Narcissistic personality disorder, and Schizotypal personality disorder. To select the most likely diagnosis, it would be necessary to review each condition in line with the symptoms exhibited by him. CLINICAL REASONING: The patient's symptoms entail a myriad of antisocial behaviors - law violations like driving under the influence, aggression towards co-workers, irresponsibility towards societal obligations, and the like starting from adolescence which persists into adulthood. Antisocial Personality Disorder (APD) typically presents with such symptoms. Conduct disorder, likewise characterized by antisocial behavior, is often diagnosed in children or adolescents and not in adults. Borderline Personality Disorder (BPD) is characterized by emotional instability, impulsive behavior, and turbulent relationships but doesn't quite vehemently line up with the patient's symptoms. Narcissistic Personality Disorder (NPD) involves an inflated sense of self-importance and lack of empathy that doesn't necessarily correlate with his narrative. Schizotypal Personality disorder (STD) includes peculiar thinking and behavior, discomfort with close relationships which is markedly different from the patient's presentation who is mentioned as 'charming' and 'interactive'. CONCLUDING: By comparing the characteristics of all the potential disorders, the patient's symptoms align most closely with 'antisocial personality disorder’. This disorder entails a pattern of manipulation, exploiting others, frequent law violations, aggressive behavior and lack of regret for such actions which coincides with the patient's behavior. RESPONSE: The most likely diagnosis based on the given symptoms and behaviour for the 20-year-old man in this scenario would be (A) Antisocial personality disorder.

**AI_B:** RESTATING THE QUESTION: A 20-year-old man has exhibited a pattern of irresponsible behavior, including getting into a car accident while drunk, getting fired for threatening behavior, and showing little concern for his financial troubles or future plans. Despite the serious nature of these problems, you find him charismatic and persuasive, and he seems genuinely intent on changing. What is the most probable diagnosis for this person's behavior? PLANNING: Personality disorders are chronic, maladaptive patterns of behavior that markedly interfere with an individual's functioning. Let's first define each of the options given: (A) Antisocial personality disorder (ASPD) is characterized by a disregard for the rights of others and a pattern of irresponsible behavior. (B) Borderline personality disorder (BPD) is characterized by emotional instability, impulsivity, risky behaviors, unstable relationships, and fears of abandonment. (C) Conduct disorder (CD) is a psychiatric disorder diagnosed in childhood that presents itself through a repetitive and persistent pattern of behavior in which the basic rights of others or major age-appropriate societal norms or rules are violated. (D) Narcissistic personality disorder (NPD) involves an inflated sense of importance, need for admiration, and lack of empathy. (E) Schizotypal personality disorder (SPD) includes symptoms such as odd beliefs or magical thinking and behavior or appearance that is odd, eccentric, or peculiar. CLINICAL REASONING: Given the patient's documented history of persistent antisocial behavior manifesting as truancy, shoplifting, running away from home, and later, threat of violence towards a co-worker, and driving under the influence, combined with his irresponsibility and disregard for the consequences of his actions, antisocial personality disorder (ASPD) seems a likely diagnosis. To note, conduct disorder is generally diagnosed in childhood and adolescence and is considered a predecessor of ASPD, but this individual is now an adult, which makes Conduct Disorder less fitting. The patient doesn't display the emotional instability or fears of abandonment typical of BPD, the deep lack of empathy and need for admiration typical of NPD, or the odd beliefs and magical thinking observed in SPD. CONCLUDING: Based on his history and behaviors, this patient's most likely diagnosis is antisocial personality disorder. However, it's essential to remember that a formal diagnosis should be made by a mental health professional following a comprehensive evaluation. RESPONSE: (A) Antisocial personality disorder.

**AI_C:** RESTATING THE QUESTION: From what I understand, the patient is a 20-year-old male that has a history of truancy, shoplifting, running away from home, and instances of violent threats towards co-workers. Moreover, he has a history of driving under the influence of alcohol and has been involved in a car accident recently. Despite having many unpaid debts and being unemployed, he shows no concern and presents no plans for seeking work or solving his financial crisis. However, he still appears charming and interactive, and even shows a sincere dedication to modifying his behavior. PLANNING: The clinical assumption that emerges from these facts is that he may have a form of personality disorder. The given choices are Antisocial personality disorder, Borderline personality disorder, Conduct disorder, Narcissistic personality disorder, and Schizotypal personality disorder. We need to evaluate each assumption in light of the patient's symptoms and behavior. CLINICAL REASONING: Antisocial personality disorder is characterized by a disregard for the rights of others, lack of remorse, deceitfulness, impulsivity, and irresponsibility - many of which seem to align with this patient’s symptoms. Borderline personality disorder involves unstable relationships, self-image, and affect, as well as impulsivity. While this patient shows signs of impulsivity, other typical traits of BPD are not evident. Conduct disorder is typically diagnosed in children or adolescents exhibiting a pattern of behavior in which the basic rights of others or societal norms are violated (e.g., shoplifting, truancy), which matches the patient’s adolescence history, but it doesn’t typically persist into adulthood. Narcissistic personality disorder is characterized by a pervasive pattern of grandiosity, need for admiration, and lack of empathy, which doesn’t seem to align with the patient’s symptoms. Lastly, Schizotypal personality disorder typically involves interpersonal deficits marked by acute discomfort in close relationships, cognitive or perceptual distortions, and eccentric behaviors, none of which seem to apply here. CONCLUDING: From the clinical reasoning process, it appears that Antisocial Personality Disorder (APD) aligns most closely with the patient's present symptoms and past history. The irresponsibility, impulsivity, lack of remorse, and disregard for societal norms are hallmark characteristics of APD. RESPONSE: Therefore, the most likely diagnosis for this patient is Antisocial personality disorder (A).

**AI_E:** RESTATING THE QUESTION: This question refers to a 20-year-old male patient who presents post-accident, possibly involving alcohol. The patient has a history of social and behavioral issues that have led to his interaction with the law, unemployment, and financial difficulties, without showing any concern or making any plans to improve his situation. He exudes charisma and gives an impression of being eager to amend his ways. The question is asking, given these facts, what is the most appropriate diagnosis. The options provided are all types of personality disorders, which are enduring patterns of inner experience and behavior that deviate markedly from the individual's culture, are pervasive and inflexible, have an onset in adolescence or early adulthood, are stable over time, and lead to distress or impairment. PLANNING: For the answer, we need to consider the history provided and compare it with the diagnostic criteria for the personality disorders mentioned in the options. Antisocial personality disorder, characterized by a history of disregarding and violating the rights of others, is one of the plausible options due to the patient's history of truancy, shoplifting, threats, and problems with the law. Borderline personality disorder presents with intense emotional instability, impulsivity, and unstable relationships, which this patient doesn't clearly show. Conduct disorder is typically a diagnosis given to children or adolescents who show generalized and serious disinterest and violation towards others' rights, not adults. Narcissistic personality disorder would involve grandiosity, lack of empathy, and a need for admiration, not adequately depicted in this case. Schizotypal personality disorder typically includes social and interpersonal deficits, along with cognitive or perceptual distortions and eccentricities of behavior which are absent in this patient. CLINICAL REASONING: Given the information provided, the evidence that the patient has repeatedly infringed and violated the rights of others, including criminal behavior, threats and disregard for laws, without exhibiting significant remorse or concern, best fits the criteria for antisocial personality disorder. It's important to note that personality disorders are typically diagnosed in adulthood, and Cluster B personality disorders specifically, like Antisocial personality disorder, frequently have an onset in adolescence. They are also consistent across time and situations, which aligns with the patient's chronic history of behavioral issues. CONCLUDING: Considering all aspects, my clinical reasoning process leads me to believe that, in this case, the young man is most likely suffering from Antisocial Personality Disorder. RESPONSE: (A) Antisocial personality disorder

**AI_A:** RESTATING THE QUESTION: As an AI, I understand that this young man is showing a long history of rule-breaking behavior, disregard for other people's rights, lack of remorse for his actions, and recurring legal problems due to driving under the influence of alcohol. Despite his problematic history, he remains charismatic and shows intent to change. We are asked to determine which personality disorder is most likely manifested in his behavior from the choices of antisocial, borderline, conduct, narcissistic, and schizotypal personality disorders. PLANNING: We can hypothesize that the most clinically relevant assumption might be his long-standing pattern of behaviors, which are an important aspect of personality disorders. All the choices given represent personality disorders, but their characteristics differ. Antisocial personality disorder is characterized by a pervasive pattern of disregard for, and violation of, the rights of others. Borderline personality disorder is typically represented by emotional instability. Conduct disorder is a repetitive and persistent pattern of behavior in which the basic rights of others or major societal norms are violated, but it is a diagnosis usually applied to children and adolescents. Narcissistic personality disorder is associated with self-centeredness, while schizotypal personality disorder involves peculiarities of thinking and behavior. Therefore, taking into account the patient's historical information along with his current behavior will be key to identifying the most appropriate diagnosis. CLINICAL REASONING: The patient displays a persistent, long-term pattern of irresponsibility, violation of the law, and lack of concern regarding his debts or employment. This behavior reflects a disregard for societal norms and the rights of others, which are indicators of antisocial personality disorder. Additionally, these symptoms have been seen since adolescence, which aligns with the criteria for this disorder as per the Diagnostic and Statistical Manual of Mental Disorders (DSM-5). The patient does not exhibit the instability of interpersonal relationships, self-image, and marked impulsivity of borderline disorder, nor the grandiosity, need for admiration, and lack of empathy characteristic of narcissism. His behavior pattern does not fit the criteria for conduct disorder, as he is over the age threshold (18 years), and he does not show the eccentric behavior, odd beliefs, or odd speech associated with schizotypal disorder. CONCLUDING: This patient's presentation fits most accurately with antisocial personality disorder. He exhibits a pattern of disregard for the rights of others, along with a pattern of irresponsible behavior since adolescence. These characteristics, combined with the lack of symptoms associated with the other personality disorders listed, narrow our diagnosis. RESPONSE: The most likely diagnosis for this patient, given his history and current behavior, would be (A) Antisocial personality disorder.

**Facilitator** The consensus among the AI responses is that the most likely diagnosis for the 20-year-old male patient in question is Antisocial Personality Disorder (APD). This conclusion is based on the patient's long-standing pattern of irresponsible behavior, disregard for societal norms and the rights of others, and lack of remorse for his actions, all of which are characteristic of APD. Despite his problematic history, he remains charismatic and shows intent to change. The patient's behavior does not align with the other personality disorders listed in the options. Borderline Personality Disorder typically involves emotional instability and unstable relationships, which are not evident in this case. Conduct Disorder is usually diagnosed in children or adolescents, and the patient is over the age threshold. Narcissistic Personality Disorder involves grandiosity, a need for admiration, and lack of empathy, which do not align with the patient's symptoms. Lastly, Schizotypal Personality Disorder involves peculiar thinking and behavior, discomfort with close relationships, which also do not apply to this patient. Therefore, the most likely diagnosis for this patient, given his history and current behavior, is Antisocial Personality Disorder. However, it's essential to remember that a formal diagnosis should be made by a mental health professional following a comprehensive evaluation.
